# Dynamic Contact Networks in Confined Spaces: Synthesizing Micro-Level Encounter Patterns through Human Mobility Models from Real-World Data

**DOI:** 10.3390/e26080703

**Published:** 2024-08-19

**Authors:** Diaoulé Diallo, Jurij Schönfeld, Tessa F. Blanken, Tobias Hecking

**Affiliations:** 1 Institute of Software Technology, German Aerospace Center (DLR), 51147 Cologne, Germany; jurij.schoenfeld@dlr.de (J.S.); tobias.hecking@dlr.de (T.H.); 2 Department of Psychological Methods, University of Amsterdam, 1018WS Amsterdam, The Netherlands; t.f.blanken@uva.nl

**Keywords:** contact networks, temporal networks, micro-level encounter modeling, human mobility models, pandemic research, Bayesian optimization

## Abstract

This study advances the field of infectious disease forecasting by introducing a novel approach to micro-level contact modeling, leveraging human movement patterns to generate realistic temporal-dynamic networks. Through the incorporation of human mobility models and parameter tuning, this research presents an innovative method for simulating micro-level encounters that closely mirror infection dynamics within confined spaces. Central to our methodology is the application of Bayesian optimization for parameter selection, which refines our models to emulate both the properties of real-world infection curves and the characteristics of network properties. Typically, large-scale epidemiological simulations overlook the specifics of human mobility within confined spaces or rely on overly simplistic models. By focusing on the distinct aspects of infection propagation within specific locations, our approach strengthens the realism of such pandemic simulations. The resulting models shed light on the role of spatial encounters in disease spread and improve the capability to forecast and respond to infectious disease outbreaks. This work not only contributes to the scientific understanding of micro-level transmission patterns but also offers a new perspective on temporal network generation for epidemiological modeling.

## 1. Introduction

The study of mobility patterns and the formation of complex contact networks remains a cornerstone in epidemic research, providing important insights into the dynamics of disease spread and informing mitigation strategies and public health policies [1,2,3,4]. This recognition has been underscored by the global COVID-19 pandemic, where the analysis of contact networks has played a pivotal role in forecasting the virus’s trajectory [5,6,7]. These networks attempt to capture the essence of human interactions, yet they often simplify the granularity of individual movements and encounters through high-level abstractions. The challenge of observing real-world contact networks directly has led to a demand for accurate simulation models that can replicate infection propagation properties of temporal networks observed in different settings.

In advancing our previous work [8], this paper extends the exploration of micro-level contact modeling by integrating sophisticated human mobility models (HMMs). These models are specifically designed to mimic the movement patterns of individuals within constrained spaces, making them ideally suited for generating temporal contact networks that reflect the nuances of specific locations. Our approach enriches the existing methodologies by utilizing temporal-dynamic networks constructed from observed real-world contacts and applying Bayesian optimization to fine-tune the parameters of our HMMs. This optimization ensures that the generated networks closely emulate infection dynamics of their real-world counterparts.

We build upon our foundation of employing simple micro-level encounter models using synthesized networks, now enhanced by the inclusion of real-world data and advanced analytical techniques, as well as more sophisticated encounter models. This progression allows for a more nuanced understanding of encounter patterns and their implications for epidemic spread. The analysis of topological network features, infection curves, and the interpretation of optimized hyperparameters represent significant parts of our methodology. These advancements can improve the accuracy of epidemic forcasting by integrating location-specific micro-level infection characteristics into large-scale infection spreading simulations. Notably, existing pandemic simulation models, such as OpenABM [9], Covasim [10], and Memillo [11], oversimplify infection propagation principles when modeling contacts and infection transmissions in confined spaces. Our approach accounts for the unique nuances and characteristics of different confined environments, enabling improved epidemic forecasting simulations, and, therefore, can support investigations on public health interventions and contact-tracing efforts. The key contributions of our paper include the following:

- The generation of temporal-dynamic networks that are based on a range of micro-level encounter models. This includes advanced HMMs designed to simulate infection properties as well as network characteristics in confined locations with high fidelity, alongside simpler models.
- The introduction of Bayesian optimization for hyperparameter selection in HMMs, a novel approach aiming at generating temporal-dynamic networks for confined spaces. This strategy focuses on accurately replicating the infection propagation dynamics observed in real-world contact networks, serving as a cornerstone in enhancing the realism and relevance of large-scale epidemic simulations.
- The employment of various network metrics for both the optimization of our models and their comprehensive evaluation, coupled with a thorough analysis that demonstrates the capability to effectively parameterize HMMs using real-world network data. This integrated approach not only validates the effectiveness of our networks in mimicking real-world phenomena but also identifies certain models as particularly well-suited for specific types of locations.

The structure of this paper is organized as follows: Section 2 sheds light on existing methods employed in micro-level encounter modeling and explores HMMs as temporal-dynamic networks. Following this, the methodology section, Section 3, then proceeds to introduce our approaches to the modeling of micro-level contacts as well as the data used in our experiments. The results section, Section 4, compares the outcomes of various techniques we have employed in the capability of generating contact networks with realistic infection propagation properties. We discuss our findings, the limitations of our approaches, as well as the potential use for future pandemic modeling in Section 5. Finally, Section 6 summarizes our work and provides an outlook on future work.

## 2. Background

In the following section, we spotlight investigations focused on the modeling of micro-level encounters as contact networks. In the context of this paper, micro-level contact modeling refers to the creation of contact networks between individuals, who encounter each other in limited environments such as supermarkets, offices, or trains. We discern the primary contributions of each methodology and analyze their respective limitations. Subsequently, different HMMs are introduced

### 2.1. Existing Approaches to Micro-Level Encounter Modeling

Two large pandemic simulation models, OpenABM [9] and Covasim [10], introduced the concept of multi-layer networks. Both models were used to investigate COVID-19 dynamics and test different intervention strategies. The multi-layer network approach uses census data to build a synthetic population on an urban scale. Contacts are generated by different models representing different types of interactions and environments in daily life. Covasim generates fully connected networks within households, small-world networks on the community and work level, and disconnected clique networks representing classes. Similarly, OpenABM employs fully connected networks at the household level, random networks for communities, and small-world networks for occupations. Both models understand the necessity for different micro-level approaches in different locations. However, they choose simplistic approaches that are grounded in small-world and random networks, which cannot accurately reflect the dynamics of micro-level interactions, proving the need for further research in that domain.

A study conducted by Klise et al. [12] harnessed mobility data to construct micro-level person encounters. This approach considers temporal intersections of individuals at locations, as well as the type of location. The authors differentiate three location types, with each being associated with three basic transmission probabilities. A final edge-transmission weight is computed by combining the location-dependent transmission risk and a score derived from the intersection time of two individuals. However, for any given location, the final transmission dynamics are solely dependent on intersection times, overlooking the spatial attributes of the location and human movement patterns.

Müller et al. [13] attach an established data-driven transportation model to an infection model, incorporating factors such as mask usage and air exchange rates specific to different location types and sub-spaces. To model micro-level contact encounters, the approach divides locations into sub-spaces of predetermined capacity, giving rise to a contact network characterized by cliques. While this leads to a sophisticated model for location-based person-to-person encounters, it requires access to mobile phone data and does not fully account for the diverse encounter patterns that different location types exhibit.

The dynamics of disease spreading in various indoor environments have also been explored by several studies using sophisticated simulation techniques [14,15,16,17]. Notably, these investigations have aimed to provide insights into transmission patterns and infection potentials in specific settings where a high amount of information is available. However, the effectiveness of such approaches relies on available and accurate information, e.g., layout, structure and architecture of the location under investigation, which limits its applicability to settings with varying spatial configurations.

Up until now, the landscape of micro-level contact modeling has been characterized by two predominant trends: network generators that mainly rely on time spent at locations as well as the associated capacities and complex physical simulations necessitating substantial data and computational resources for agent-based modeling. While the former overlooks important interaction dynamics, the latter is resource- and data-intensive and may not be feasible in many scenarios. In the methodology section, Section 3, we outline approaches to model location-specific encounter patterns capable of capturing significant interaction dynamics while maintaining low computational cost.

### 2.2. Human Mobility Models

Accurately modeling human mobility is crucial in understanding and forecasting the dynamics of infectious disease spread, but also in other domains such as communication networks and urban planning. The development and refinement of mobility models provide essential insights into the complex patterns of human movement. These models serve as the backbone for simulating scenarios that reflect real-world behaviors, potentially addressing issues of micro-level encounter models recently used in epidemic simulations. In the following, four HMMs are presented, which were used in our study:

**Random Waypoint (RWP)** [18] is a foundational mobility model where individuals randomly select destinations and travel towards them at constant speeds that are drawn from a uniform distribution $U(v_{min},v_{max})$. After reaching a destination, a pause may occur before the process repeats. The pause duration is drawn from a uniform distribution $U(t_{p,min},t_{p,max})$. *RWP* is valued for its simplicity and provides a baseline for understanding movement patterns in various contexts.

**Truncated Levy Walk (TLW)** [19] models human movements as a mix of short, frequent trips and rare, longer journeys. The trip length *l* is drawn from a truncated power law with shape $\alpha_{l}$ and a maximum value of $H_{l}$. Similarly, pause times between flights are drawn from a truncated power law with shape $\alpha_{t_{p}}$ and maximum value $H_{t_{p}}$. This model accurately describes the heavy-tailed, power-law distribution of human trip lengths, offering insights into mobility over large areas and its implications on epidemic spread.

**Spatio-Temporal Parametric Stepping (STEPS)** [20] abstracts human mobility with a focus on spatio-temporal preferences. Nodes exhibit preferential attachment to favorite locations, by structuring the location into sub-spaces. Each agent chooses a default location $Z_{default}$; after a certain waiting time, drawn from a Pareto distribution $P(t_{p,min}=1,\alpha_{t_{p}})$, a trip is made where a trip distance *d* is drawn from

(1) $$P\left(D=d\right)=\frac{\xi }{{\left(1+d\right)}^{k}}.$$

While ξ is a normalization constant, the parameter *k* reflects how strong nodes are attached to their default location and their close surroundings. At k=0, preferential attachment vanishes completely and nodes choose their sub-spaces randomly. Then, a random sub-space $Z_{i}$ from all sub-spaces that fulfill $d(Z_{default},Z_{i})\leq d$ gets selected and the node travels to its location with a speed drawn from a uniform distribution $U(v_{STEPS,min},v_{STEPS,max})$. *STEPS* captures essential characteristics of human mobility, such as preferential attachment to close-distance areas, and the ability to model small-world network structures inherent in human interactions.

**STEPS with RWP** [20] combines the *STEPS* model and the *RWP* model. Instead of nodes not moving within their sub-space, they perform movement according to the *RWP* model while waiting for their next trip.

These models serve as crucial tools for our study, enabling the simulation of a wide range of human mobility behaviors and their impact on epidemic dynamics. By studying these models in the context of contact networks based on micro-level encounters and propagation dynamics, we can better understand how diseases spread and develop more effective strategies for controlling outbreaks.

### 2.3. Temporal-Dynamic Contact Networks

Temporal-dynamic networks serve as a sophisticated framework that reveals the ever-changing nature of interactions among individuals [21]. In contrast to static networks, which offer a snapshot of connections, temporal-dynamic networks capture the intricate evolution of relationships over time. This real-time depiction introduces a higher level of realism, as interactions are not treated as fixed entities but rather as dynamic occurrences. Temporal-dynamic networks prove invaluable in epidemiological studies, as they grant insights into the spread of diseases over time [22]. By incorporating time-varying edges, these networks portray the varying transmission potentials at different stages of an epidemic. This precision empowers researchers and policymakers to devise strategies for disease containment and control more effectively.

Temporal-dynamic networks present interactions as evolving sequences, not mere snapshots [23]. While dynamic networks are gaining traction in pandemic research, many studies still rely on static networks due to their computational simplicity. Although static networks can suffice when disease dynamics align with network changes, they can introduce biases. Such biases arise when aggregating variable dynamic contacts, leading to misrepresentations in potential infection paths. It is debated that static networks might intensify infection dynamics. On the other hand, some cases are known where temporal correlations accelerate the dynamics of stochastic processes in dynamic networks compared to their static equivalent. In [24], infection spreading simulations were performed on an empirical temporal network of sexual interactions to investigate the spreading of sexually transmitted infections. Their findings suggest that, especially in the early pandemic stage, temporal correlations in the network accelerate infection dynamics leading to higher outbreak sizes, compared to different variations of static network representations.

## 3. Methodology

This section outlines the core methodologies that support our study of micro-level interaction modeling via temporal-dynamic networks. Initially, the infection propagation model used throughout this study as well as the real-world network data are presented. We then proceed to describe methods for modeling micro-level contacts. This spectrum includes simplistic, naive approaches as foundational baselines, alongside more complex techniques that make use of HMMs.

### 3.1. Susceptible–Infectious–Recovered Model

The overall goal of this study is the generation of realistic contact networks for confined spaces, which reflect the infection propagation properties of their real-world counterparts. For the parameterization of the HMMs used in this study, as well as the comparison of resulting contact networks, we employed the Susceptible–Infectious–Recovered (SIR) model [25]. The SIR model is a well-established compartmental model used to analyze the spread of infectious diseases within a population. It divides individuals into three compartments: susceptible (S), infectious (I), and recovered (R). The SIR model tracks the transitions of individuals between these compartments based on their interactions and the disease’s transmission dynamics. For our evaluation, we utilized a temporal-dynamic SIR model implemented using the Tacoma framework (https://github.com/benmaier/tacoma, accessed on 30 July 2024). Tacoma provides a versatile platform for studying epidemic spreading and other dynamical processes on networks utilizing the Gillepsie algorithm [26]. We let the epidemic spreading simulations run for a simulated period of 35 artificial days. During this time, we monitored the progression of the infection within the population and observed how different modeling approaches influenced the spread of the disease. This SIR-based evaluation allowed us to gain insights into the impact of micro-level encounter modeling on the topological properties of contact networks and the resulting epidemic dynamics.

To ensure adequate infection dynamics in our simulations, we set a uniform recovery rate across all networks, with 1/γ=7 days, indicating that an infected node typically recovers within a 7-day period. By selecting β values that would achieve roughly 20% of infectious nodes at the infection peak, we could maintain a robust dynamic across the experiments. The chosen β values for the networks in our study—namely, high school, primary school, office, and supermarket—were 0.007, 0.0013, 0.013, and 0.075, respectively. Using varied transmission rates allowed us to simulate realistic infection dynamics by accounting for the distinct nature of each network while ensuring that the desired infection peak was met.

### 3.2. Dynamic Network and Mobility Data

In this work, we utilized two distinct data sources to inform and test our models: temporal-dynamic networks from socio-patterns and supermarket mobility data. We will now detail these sources and discuss any associated technical limitations. It is important to note that, to conduct comprehensive SIR simulations across several days, we addressed the challenge posed by the availability of accurate long-term mobility data. Our approach involved stacking the temporal contact network data from single days to simulate a continuous span of 35 days. This step was necessary to ensure a fair comparison between different locations, as the original period of the temporal network datasets used in this study varied heavily. In environments where individuals typically have assigned, consistent interactions (e.g., most offices and schools), this method provides a reasonable approximation. While this approach may not fully capture long-term fluctuations, it still allowed us to create contact networks with a certain extent in infection dynamics and identify infection potential specific to confined spaces. Conversely, in locations with high variability in individual movement and interactions, such as supermarkets, the infection dynamics likely deviate more from actual dynamics. We discuss this further in the context of our supermarket network below.

#### 3.2.1. Socio-Patterns Network Data

We incorporated three real-world datasets for specific settings: a high school network [27], a primary school network [28], and an office network [29]. These temporal-dynamic networks consist of interactions between individuals over different periods. The datasets do not record exact arrival, departure, and duration times; these must be inferred from the timing of the first and last interactions of each node. This means that, for each person, the arrival time is marked by the occurrence of the first edge, and the departure time is set by when their last interaction ends. Due to the experimental setup noted in these studies, the data capture only face-to-face contacts that occur within a range of up to 1.5 m. Our analysis concentrated exclusively on the recorded physical encounters included in the datasets. We adopted the temporal resolution of 20 s given by the empirical networks. For the purpose of this study, we will refer to *real-world networks* as networks which have been constructed from observed real-world [27,28,29].

#### 3.2.2. Supermarket Network Data

In [30], encounters of supermarket visitors were recorded during the COVID-19 pandemic. In addition to encounters, the exact arrival and departure times are provided as well. The data encompass approximately five hours of encounters.

In contrast to the socio-patterns datasets, the supermarket dataset was created using ultra-wideband technology instead of radio devices, providing significantly higher sensitivity. Despite this improvement, individuals still wore the devices in front of their bodies, leading to potential limitations in detecting interactions when people were positioned behind one another. As a result, we assumed a similar field of view as in the other datasets, indicating that the devices can detect encounters within this range. To align with the temporal resolution of the socio-patterns networks, we aggregated the contacts of the supermarket network into 20-s intervals.

In our study, which examines the spread of infections within confined spaces, supermarkets were not ideal locations for analysis due to the infrequent visits by individuals, which inhibits the initiation of infection dynamics. To mitigate this limitation, we also used receiver IDs assigned to participants during the experiment to keep track of nodes over multiple days (as explained above, we stacked single days of network data to longer time spans). This approach allowed us to utilize the supermarket network as a proxy for locations that attract the same group of visitors but exhibit highly random movement patterns compared to schools or offices. In general, locations with infrequent individual appearances are not conducive to modeling the propagation of infections over extended durations, and are, therefore, more effectively integrated into large-scale simulation frameworks. Nevertheless, our analysis benefitted from including the supermarket case, as it introduced a distinct nature of interactions compared to socio-patterns networks, allowing us to explore the adaptability and optimization of our models for differently characterized networks.

### 3.3. Micro-Level Contact Modeling

This section introduces various micro-level contact models. Initially, we recapitulate naive approaches from our prior work [8], before advancing to innovative contact modeling techniques, which leverage HMMs and Bayesian optimization.

While aggregated mobility data may be available, detailed micro-level movements are typically not available, leaving a gap in accurately modeling the nuanced interaction patterns that influence infection dynamics. By bridging this gap, micro-level models can enhance the precision of infection forecasting. In the context of this study, micro-level encounter modeling aims to generate temporal networks that reflect infection dynamics and certain network properties of observed real-world temporal networks. All models discussed are designed to input arrival and departure times, producing temporal contact networks that, while maintaining consistent overall node counts, differ in the number and duration of edges due to varying movement patterns.

Real-world datasets used in this study offered an actual temporal network as a baseline for validation, essential for the parameterization of HMMs. As already pointed out, we inferred arrival and departure times in these cases based on a node *i*’s initial and final edge appearance, assuming that nodes were not leaving the location between contacts.

Surprisingly, a significant number of brief edge durations could be observed when nodes that have been isolated for longer periods, such as two hours, were completely removed from the network. Apparently, nodes spent a considerable time without any contact recorded, even in school environments. This is likely due to the face-to-face nature of the empirical networks. In [31], a similar experimental-technical infrastructure was employed as in the empirical networks used in this study, but focusing on temporal networks at scientific conferences. The study found that contacts were rare during presentations, even in crowded rooms, because attendees generally do not face each other. This implies that, in other environments, such as offices and high schools, close proximity alone does not guarantee that contacts are recorded by radio devices. To ensure that our models accurately represented this aspect, we chose to model nodes continuously, from their first appearance to their last in the empirical network, without removal, even if they showed no contacts for prolonged periods.

#### 3.3.1. Naive Micro-Level Encounter Models

**Baseline approach (BASE):** our baseline approach delivers the most simplistic and intuitive way to build contact networks from the arrival and departure times of individuals at certain locations. A similar approach was described by [12]. In essence, this method leverages mobility data and individual-specific time allocations at specific locations to compute intersecting time frames between individuals, subsequently constructing contact networks. Individuals present at the same location are linked by edges in a contact network. Transforming this concept into a temporal-dynamic network, we established edges connecting pairs of individuals who coincide at a given point in time within the same location (see Section 2.3). Under this premise, our approach assumes an equal likelihood of infection for any pair of individuals who share the same duration of stay at a location. In other words *BASE* constructs a fully connected network between all nodes active at time *t*. Additionally the contact intensity *w* influences the weight of an edge. This parameter *w* is assumed to be constant for all edges and is determined by the type of location, e.g., locations with a lot of social interactions like kindergartens or cafes are assumed to have a higher *w* value than libraries or supermarkets. A noticeable difference between our approach and the approach suggested by [12] is that, in our case, nodes are always connected to all other active nodes, while, in their case, the number of contacts was capped at 20. While, in big locations, both approaches generate very different networks, they are identical for small locations where the number of nodes stays below 20 for the majority of the time. This simplified framework formed the foundation of our exploration, serving as a reference point against which we compared our more intricate modeling techniques.

**Random graph-based approach (RAND):** in our random graph-based approach, similar to [9], every possible edge, meaning any nodes *i* and *j* present at the location at time *t*, is selected with probability $p_{RAND}$. Additionally, a contact duration is drawn from a Pareto distribution $P(t_{cd,min}=1,\alpha_{cd})$. Contacts, therefore, have a minimum duration of a one-time step and follow a power law determined by the shape parameter $\alpha_{cd}$. This distribution accounts for the variable nature of interaction durations, resulting in a dynamic and realistic representation of human encounters when the recurrence of contacts is completely random. A possible application would be in locations where interactions form mainly due to uncorrelated movement instead of social relations, like in supermarkets, where the case of two individuals being nearby for the entire shopping trip is rather unlikely; however, frequent but short contacts are to be expected.

**Clique-based approach (CLI):** we advanced the clique-based strategy of [13] for the purpose of micro-level encounter modeling. To construct cliques, we utilized a combination of spatial dynamics and contact patterns. First, individuals are assigned to sub-spaces within the location, with the parameterizable Npps mirroring the number of individuals per space. Whenever a node is introduced into the location, it stays in its space and forms connections to all nodes present in this space, forming tight cliques. To allow contacts between cliques at every time step, a node changes its space with probability $p_{clique}$ for a duration that is drawn from a normal distribution N(μ,σ). Afterward, the node returns to its default space.

**Clique-based approach with random substructure (CLI+RAND):** cliques from *CLI* can be imagined as classrooms, offices, or apartments in residential buildings. Large values for Npps will generate a high number of contacts, e.g., a classroom with 30 students already generates 435 edges, at every time frame, due to their fully connected nature. To lower the density of the clique networks and allow for edge changes, we added our *RAND* approach to sit on top of the clique structure. Contacts within cliques are now randomly sampled according to the procedure explained in *RAND*. This leads to the formation of cliques with an adjustable density, where individuals have pronounced edges connecting them within the clique, reflecting intensive interactions. In contrast, connections outside the clique are rare, mirroring more sporadic or distant interactions. The underlying idea of this approach is to encapsulate the nuanced interplay between spatial arrangements and interpersonal encounters.

#### 3.3.2. Human Mobility-Based Micro-Level Encounter Models

To build temporal contact networks from HMMs, we used an open-source implementation of RWP and TLW (https://github.com/panisson/pymobility, accessed on 30 July 2024) that follows the model description provided in Section 2. The STEPS model was integrated with RWP to form the combined STEPS+RWP model, as detailed in Section 2.2. Since all models need a confined area for nodes to walk in, we built synthetic locations according to our empirical networks. We therefore inferred the capacity *C* of each location. For the primary school, the high school, and the office networks, we assumed the capacity of the location to be equal to the number of participants in the experiment. The capacity for the supermarket network was defined as the peak number of active nodes across all time steps, which resulted in a capacity of 44. To determine the area based on a location’s capacity, we utilized values for location-dependent space per person from [13], denoted as ρ. We developed a quadratic surface based on these values, calculating the area *A* as A=C×ρ. For the STEPS-based models, we additionally sub-structured the area into $\left\lceil \sqrt{N_{V}/Npps}\right\rceil$ sub-spaces in the horizontal and vertical direction, where $N_{V}$ denotes the number of nodes (see Table 1). This ensures a uniform distribution of sub-spaces across the entire area, resulting in some sub-spaces potentially containing fewer nodes than the default Npps.

In all HMM-based approaches, we continuously tracked node movements during the simulation. A contact between two nodes *i* and *j* is generated if both contact conditions

(2) $$\mathrm{Cond}.1:0\leq d\left({\vec{r}}_{i},{\vec{r}}_{j}\right)\leq d_{\max},$$

(3) $$\mathrm{Cond}.2a:\arccos\left(\frac{{\vec{v}}_{i}\cdot {\vec{r}}_{ij}}{∥{\vec{v}}_{i}∥\cdot ∥{\vec{r}}_{ij}∥}\right)\leq \frac{\varphi^{*}}{2},$$

(4) $$\mathrm{Cond}.2b:\arccos\left(\frac{{\vec{v}}_{j}\cdot {\vec{r}}_{ji}}{∥{\vec{v}}_{j}∥\cdot ∥{\vec{r}}_{ji}∥}\right)\leq \frac{\varphi^{*}}{2},$$

are fulfilled. Cond.1 ensures that, for nodes positioned at $\vec{r_{i}}$ and $\vec{r_{j}}$, the distance between them must be smaller than a specified maximum distance $d_{max}$ to result in an edge. To calculate distances between all nodes at every time step, we used the well-known kd-tree algorithm, utilizing the standard Euclidean distance. Secondly, we wanted to determine whether the lines of sight of both nodes aligned or not, accounting for the experimental design outlined in Section 3.2.1. Therefore, we defined their line of sight vectors, $\vec{v_{i}}$ and $\vec{v_{j}}$, which are always parallel to the latest movement. For all nodes that pass condition Cond.1, the angles between their lines of sight and the connecting vector $\vec{r_{ij}}=\vec{r_{j}}-\vec{r_{i}}$ are calculated. If those angles are smaller or equal to one-half of their field of view $\varphi^{*}$, then conditions Cond.2a and Cond.2b are fulfilled, and a contact between nodes *i* and *j* is generated. Following the conditions from the original studies outlined in Section 3.2, we set the field of view to 120 degrees, a typical value for the human binocular field of view, and the maximum contact distance to 1.5 m. The timescale of our simulation was one second. To align with the timescale of the empirical networks, we established a contact if nodes met at least once within a 20-s window. Consequently, a contact was considered ended if nodes lost contact for at least 20 s.

#### 3.3.3. Bayesian Optimzation for Hyperparameter Selection

To perform hyperparameter optimization, we used the Optuna framework described in [32]. Optuna employs advanced Bayesian optimization algorithms to identify a set of parameters from a specified search space that minimizes a designated objective function. A comprehensive table detailing all tuned parameters and their respective ranges can be found in Appendix A.1. To evaluate the error generated by a specific model, we focused on metrics that assess the similarity between the infection dynamics of the empirical network $G_{e}$ and the modeled network $G_{m}$. The infection dynamics were calculated as outlined in Section 3.1. Here, $I_{m}$ and $I_{e}$ denote the number of infected nodes for $G_{m}$ and $G_{e}$, respectively. We measured both the difference in infection peaks, denoted as $\Delta I_{max}$, and the timing difference of these peaks, expressed as $\Delta T_{I_{max}}$, using the following formula:

(5) $$\Delta I_{max}\left(I_{m},I_{e}\right)=\frac{1}{N_{V}}\left|\max_{t}\left\{I_{e}\left(t\right)\right\}-\max_{t}\left\{I_{m}\left(t\right)\right\}\right|,$$

(6) $$\Delta T_{I_{max}}\left(I_{m},I_{e}\right)=\left|\frac{\underset{t}{\arg\max}\left\{I_{e}\left(t\right)\right\}-\underset{t}{\arg\max}\left\{I_{m}\left(t\right)\right\}}{\underset{t}{\arg\max}\left\{I_{e}\left(t\right)\right\}}\right|,$$

Such that a model achieving a low value for $\Delta I_{max}$ closely replicates the peak number of infections observed in the empirical network. When a network yields a small value for $\Delta T_{I_{max}}$, it suggests that the timing of the peaks in both the model and the empirical network align closely, demonstrating that the model effectively captures the empirical infection dynamics. In general, topologically different networks can generate very similar infection dynamics. To address this, we also took into account the overall number of edges generated as well as the similarity in the contact duration distributions. The relative difference in the total number of edges between two networks is defined as

(7) $$\Delta N_{E}\left(G_{m},G_{e}\right)=\left|\frac{N_{E,\;G_{e}}-N_{E,\;G_{m}}}{N_{E,\;G_{e}}}\right|,$$

where $N_{E,\;network}$ is calculated using

(8) $$N_{E,\;network}=\sum_{i,j,t}a_{i,j,\;network}\left(t\right).$$

To assess the similarity between two contact duration distributions, we utilized the well-known Kolmogorov–Smirnov (KS) test. The KS test quantitatively determines if two underlying one-dimensional probability distributions differ significantly. We computed the difference in the contact duration distribution with

(9) $$\Delta t_{cd}\left(G_{m},G_{e}\right)=KS\left(G_{m},G_{e}\right).$$

After conducting explorative experiments with various weights and observing the typical value spectra for each metric, we defined the objective function L(mod) with the following weights:

(10) $$L\left(G_{m},G_{e}\right)=5\cdot \Delta I_{max}\left(I_{m},I_{e}\right)+3\cdot \Delta T_{I_{max}}\left(I_{m},I_{e}\right)+2\cdot \Delta N_{E}\left(G_{m},G_{e}\right)+\Delta t_{cd}\left(G_{m},G_{e}\right).$$

All models, except for *BASE*, in our framework, are stochastic. Consequently, the outcome of $L(G_{m},G_{e})$ not only depends on the parameters selected from the search space but also varies around a mean value. Additionally, SIR runs are stochastic and need to be executed multiple times. To balance the stochastic variations of the SIR runs and the network construction, we generated 20 network realizations for a given set of parameters and conducted 250 SIR runs per network, a number of runs that resulted in stable infection peaks during our tests. The mean value of all $L(G_{m},G_{e})$ values (1 for each network realization) was considered as the objective function value for that trial. For each model, a total number of 150 trials was computed by scanning the search space for the optimum parameter set.

Due to the stochastic nature of the model, the final parameter set can still yield slight differences in the final objective function value. To account for this, we conducted a final test with 21 networks generated using the optimal parameters. The definitive value for $L(G_{m},G_{e})$ was determined as the median among these 21 networks. All model evaluations within our experiments were applied to these resulting median networks, including final SIR runs with $10\times N_{V}$ iterations. Appendix A.1 lists all the model parameters used in the Bayesian optimization process.

## 4. Results

This section presents findings from experiments that examined the impact of different contact patterns on infection dynamics across various scenarios. We used HMMs and optimized them according to our proposed methodology. This approach enabled us to construct contact networks that replicate infection dynamics and network characteristics observed in real-world settings. The results were then used to compare the cost values for different models, with correlations drawn to the corresponding SIR dynamics. We also analyzed the network properties and outlined the parameters derived from the optimization process.

Following the methodologies described in Section 3.3.2 and Section 3.3.3, we fine-tuned the parameters of HMMs. We performed SIR simulations, running a total of ten times the number of nodes for each network, to ensure statistically robust outcomes. We used the high school, primary school, office, and supermarket networks, introduced in Section 3.2.1 and Section 3.2.2.

**Figure 1:**
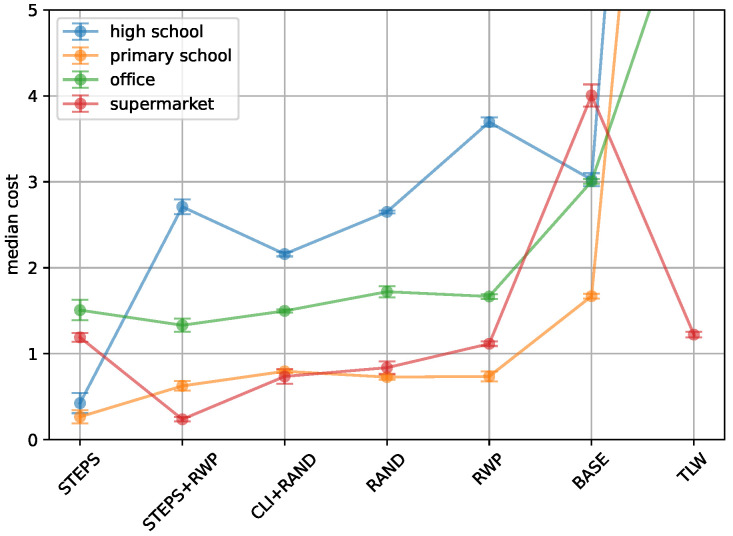
**Comparison of cost value for different encounter models.** The x-axis shows the encounter model, while the y-axis depicts the median cost. The results cover an office, a high school, a primary school, and a supermarket. The median cost is used to account for variability in the stochastic processes involved in generating the networks (see Section 3.3.3).

Figure 1 presents the costs associated with each method as derived from the objective optimization function. The *STEPS* and *STEPS+RWP* approaches consistently achieve the lowest costs across most types of locations, followed by *CLI+RAND* and *RAND*. Notably, for *TLW*, *RWP*, and *BASE*, performance varies significantly with location type. For instance, *TLW* achieves costs lower than 1.5 for the supermarket location but exceeds a cost of 10 at other locations. This variability highlights the location-dependent performance of models, a topic we will explore further in Section 5. Overall, the high school network presents the greatest challenge for the tested models, followed by the office network. Conversely, the primary school and supermarket networks yield the lowest costs across all tested models.

Although inherent randomness in both the SIR evaluations and our network models may lead to deviations from the median in the final 21 runs, the results demonstrate reliable consistency. This consistency is evidenced by the 95% confidence intervals, which do not exceed 13% above or below the median value. This indicates that our model’s performance is stable, as 95% of the expected cost values fall within these error bars shown in Figure 1.

Infection dynamics are driven by the transmission probability parameter β and recovery probability parameter γ. To verify that our top model, *STEPS*, performs well irrespective of these parameters, we examined its performance across various β and γ combinations. Specifically, we tested seven different β values for each location, combining each β value with three γ values (1/γ= 4 days, 1/γ= 7 days, and 1/γ= 10 days) to reflect recovery on average after 4, 7, and 10 days. Observing only minimal fluctuations in L, the results showed good adaptability to different β and γ values. For detailed results, see Appendix A.4.

Table 2 details the parameters of the *STEPS* and *STEPS+RWP* models, as discussed in Section 3.3.2, optimized using the Bayesian optimization strategy detailed in Section 3.3.3. For the high school network, the *STEPS* model yields an Npps value of 27, compared to 39 for the primary school, suggesting a higher density of individuals per unit space in the primary school. For the *STEPS+RWP* model, the Npps for the high school is 21, while it is 22 for the primary school, showing no pronounced difference.

The attractor strength *k* for the primary school is greater in the case of the *STEPS* model, showing a value of 9.974 for the primary school and 4.387 for the high school, while for *STEPS+RWP*, the primary school has an attractor strength of 7.870 compared to 8.128 for the high school. While, for *STEPS+RWP*, again, no strong difference is observable, the attractor strength of *STEPS* suggests that individuals in primary schools are more tightly bound to specific spaces and less likely to change the space compared to those in high schools. Besides Npps and *k*, the models show similarities in the $\alpha_{t_{p}}$ value, which determines the shape of the Pareto distribution for pause times. A larger $\alpha_{t_{p}}$ will result in more movement, as the pause times between movements are decreased. As a consequence, the higher the $\alpha_{t_{p}}$ value, the more short-term contacts occur. Both models show higher values for the primary school compared to the high school. This indicates that the likelihood of short-term contacts is somewhat greater in the primary school setting. The *STEPS* model, especially, which shows the lowest cost for most locations, reflects the distinct characteristics of high schools and primary schools by accounting for shorter, more frequent contacts in the primary school, along with a tighter adherence of primary school children to their default spaces.

In the supermarket scenario, the Npps values for both *STEPS* and *STEPS+RWP* are on a similar scale, with 20 for *STEPS* and 24 for *STEPS+RWP*. However, the attractor strengths between the two models differ strongly. *STEPS* has an attractor strength of 2.387, while *STEPS+RWP* uses a much stronger value of 9.161. Despite this, the $\alpha_{t_{p}}$ values are similarly high, with *STEPS* at 2.887 and *STEPS+RWP* at 2.172. Furthermore, the parameter $t_{p,max}$, used only in *STEPS+RWP*, is considerably lower in the supermarket scenario (24 s) compared to other locations, where it exceeds 1000 s. This constant movement causes individuals to have very short inter-contact periods, while also experiencing very brief contacts. Besides $t_{p,max}$, $\alpha_{t_{p}}$ clearly exceeds for the supermarket compared to the other locations. With $\alpha_{t_{p}}=2.887$ for *STEPS*, and $\alpha_{t_{p}}=2.172$ for *STEPS+RWP*, both models introduce a high number of short-term contacts. In contrast, both models show $\alpha_{t_{p}}$ values ranging from 0.1 to 0.8 for all other locations. These differences align with the nature of the underlying location characteristics: supermarkets are characterized by frequent short-term encounters, while schools and offices tend to have longer-lasting interactions. In the office network, the *k* values are relatively low, with 2.881 for *STEPS* and 5.120 for *STEPS+RWP*. The parameter $\alpha_{t_{p}}$ is notably higher in this setting for *STEPS*, at 0.768, compared to other locations. This suggests that the *STEPS* approach indicates a higher frequency of short-term contacts in offices than in school environments, but significantly less than those observed in supermarket scenarios. The *STEPS+RWP* model has an $\alpha_{t_{p}}$ value of 0.346, positioning it between the values found in high schools and primary schools.

**Figure 2:**
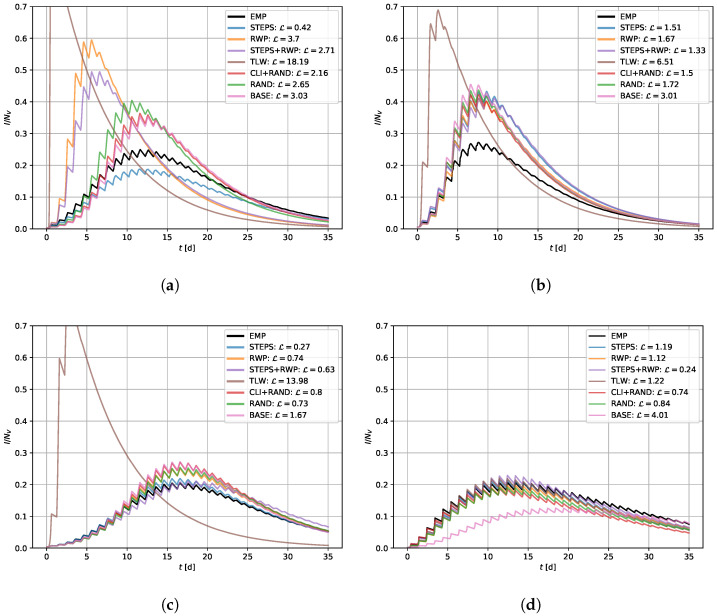
**Comparison of SIR curves for different models and empirical networks (EMPs) across various locations: high school, office, primary school, and supermarket.** The x-axis represents time in days (t[d]), and the y-axis represents the proportion of infected nodes ($I/N_{V}$). The legend includes cost values associated with each approach, and EMP represents the ground truth. (**a**) High School; (**b**) Office; (**c**) Primary School; (**d**) Supermarket.

To explore the correlation between the computed costs and the SIR curves generated by the parameterized models, Figure 2 illustrates the SIR curves for the temporal contact networks derived from various encounter models. Each subplot’s legend indicates the corresponding cost for each model. Models with a cost of up to 1 demonstrate precise replication of infection dynamics in terms of both timing and extent, e.g., *STEPS* in the primary school network scenario. A cost ranging from 1 to 2 still indicates some similarity to the infection propagation properties of the real-world network, such as *STEPS* in the case of the office network, yet deviations from the baseline infection dynamics can be observed. Costs exceeding higher values tend to result in infection dynamics that significantly diverge from real-world dynamics, as observed for *RWP* in the high school scenario. Nonetheless, our methodology proved effective, as we were able to apply Bayesian optimization and HMMs to deploy encounter models that generate temporal networks reflecting properties of realistic, location-specific infection dynamics.

**Figure 3:**
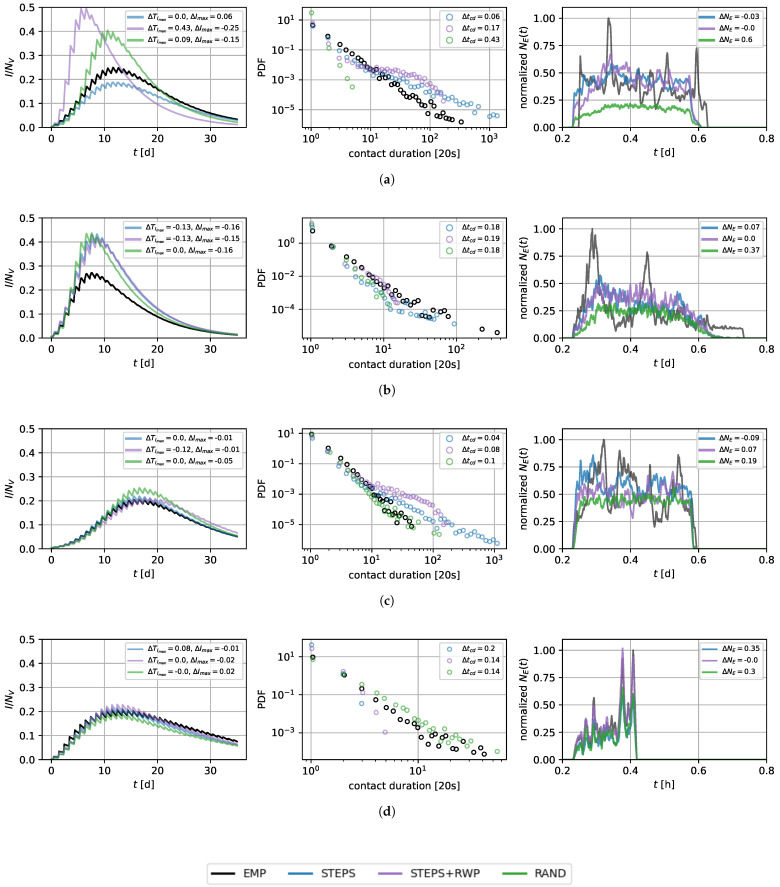
**Comparison of *STEPS* and *STEPS+RWP* with the empirical network (EMP).** The leftmost column shows the SIR curves, with the proportion of infected individuals ($I/N_{V}$) on the y-axis and time in days (t[d]) on the x-axis. The middle column displays the probability density function (PDF), with the contact duration on the x-axis. The rightmost column depicts the normalized edge counts ($N_{E}\left(t\right)$), with time in hours (t[h]) on the x-axis. All depicted edge counts are smoothed over 20 time steps. (**a**) High School; (**b**) Office; (**c**) Primary School; (**d**) Supermarket.

Figure 3 illustrates the outcomes of applying the parameterized models *STEPS*, *STEPS+RWP*, and *RAND* to the four selected locations, focusing on not only SIR curves but also the probability density functions of contact durations and edge counts in both the generated and real-world networks. Contact durations and edge counts for all models can be found in Appendix A.2 and Appendix A.3. The *STEPS* approach successfully produces temporal contact networks that emulate real-world SIR curves, with the greatest deviations observed for the office network. The outcome for *STEPS+RWP* is comparable, though it also shows a significant deviation of the SIR curve and contact durations in the high school case. The *RAND* approach mostly captures the temporal peak in infection but results in a higher number of infections across most locations. In fact, the *RAND* approach-based networks tend to underestimate edge counts but result in a network topology associated with higher infection dynamics than the real-world counterpart. This, again, underscores the critical role of network topology in shaping SIR dynamics, beyond mere connectivity levels.

Although our models do not yet account for the exact temporal distributions of edge counts (as observed in Figure 3), they can already produce realistic infection dynamic properties. We anticipate that incorporating additional network measures will further improve our modeling capabilities. An analysis of this limitation is provided in the discussion section. Notably, in the supermarket scenario, where the arrival and departure times of each individual were available (see Section 3.2.2), our models successfully reflected the temporal aspects of the edge count distribution.

In regard to the contact duration distributions, the office and supermarket networks especially show the greatest deviations from the ground truth. *STEPS* manages to replicate these distributions for the primary school and the high school locations to a large extent. Interestingly, while the *RAND* model generally underestimates the edge counts, the contact duration distributions are varying, with an overestimation of contact durations in the supermarket case, and a strong underestimation for the high school.

## 5. Discussion

The results of our study demonstrate that employing HMMs and Bayesian optimization can effectively create dynamic temporal networks that mirror infection dynamics and certain network characteristics of temporal-dynamic networks constructed from observed real-world contacts. The high degree of interpretability of the optimized hyperparameters, coupled with the ability to control model parameters, underscores the robustness and utility of our approach. By incorporating detailed, micro-level encounter data, our methodology contributes significantly to enhancing the reliability and precision of infection forecasting models. This is particularly crucial for improving responses and strategies in future pandemics, ensuring that interventions are both timely and based on robust, data-driven insights. Our results not only validate the effectiveness of our modeling approach but also highlight the importance of granular contact networks for accurately predicting the spread of infectious diseases across various locations. Overall, the availability of fast, simple, and interpretable models is essential for rapid response in pandemic situations, a need our study addresses. These models can easily be parameterized and quickly deployed, providing effective solutions, even when data are scarce.

The examination of the optimized parameters, detailed in Table 2, allows for interpretability. One notable observation is that the model incorporates parameters that align with real-world characteristics of different locations. For instance, primary schools typically have fixed classrooms, while high schools often feature dedicated rooms for specific subjects. Additionally, the likelihood of interaction with individuals from other classes might be higher, particularly in courses like language classes where class assignments may vary. This tendency is reflected for all parameters, the attractor strength *k*, the Pareto distribution shape value $\alpha_{t_{p}}$, and the Npps value. Nevertheless, it is conceivable that a different set of parameters could theoretically produce similar infection dynamics. Furthermore, this interpretation is highly driven by the characteristics of the reference locations used, which, in our case, are based in western countries. Thus, to achieve a deeper understanding and validation, further evaluation and additional experiments are essential.

As discussed in Section 4, the model representation of a supermarket exhibited a significant difference in the *k* parameter between simulations using *STEPS* with and without the RWP component. Specifically, while *STEPS* modeled the supermarket with a *k* of 2.387, *STEPS+RWP* utilized a value of 9.161. The likelihood of individuals staying within their default space is, therefore, higher with the *STEPS+RWP* model compared to *STEPS*. This generally creates a more distinct clique structure, where nodes in the same space are more likely to connect. However, both models seem to address the nature of short-term contacts by setting the parameter $\alpha_{t_{p}}$ so high that all individuals are effectively in constant motion. This approach leads to frequent but short-term contacts. In this case, it does not matter whether these contacts occur within a single space or across different spaces, as both models can create temporal network topologies that reflect the properties of the ground truth network. Nevertheless, all interpretations must be approached with caution due to the high degree of interdependence among the various parameters.

When comparing edge counts generated by different models across various locations to the real-world counterparts, the supermarket scenario exhibited the highest level of similarity. This is attributed to the availability of precise arrival and departure times, as highlighted in Section 3.2.2. The accuracy of this information is significant because individuals can only encounter each other when they are present at the same location. For the high school, primary school, and office networks, however, the arrival and departure times are inferred from the first and last edges in the data. Conversely, in the supermarket scenario, knowing the exact number of individuals who might meet leads to more accurate modeling of edge counts over time. We assume that the availability of exact arrival and departure times would enhance the performance for other real-world networks, resulting in more consistent network characteristics and SIR outcomes.

The *RWP* and *TLW* mobility models generally align with the concept of random movement, where individuals randomly select destinations, move towards them, and then pause for varying durations before repeating the process. This characteristic can explain why these models delivered moderate results in our experiments with the supermarket network, yet failed to capture the dynamics of other real-world networks like offices, high schools, and primary schools. In the supermarket scenario, individuals often move randomly between aisles, making the *RWP* and *TLW* models somewhat effective at capturing these patterns. However, these models lack the concept of attachment to specific locations, a key feature in environments like offices, high schools, and primary schools, where people tend to stay in defined areas for extended periods. The *STEPS* approach, which emphasizes a stronger attachment to certain spaces within a location, better represents these scenarios. Regarding the cost function, the *STEPS+RWP* model showed optimal performance in the supermarket network, highlighted by a close match in network properties and SIR curves. However, although the model managed the primary school network adequately, it struggled to accurately simulate the high school network. On the one hand, the model demonstrated strong performance in two very different locations: the primary school and the supermarket. This adaptability can be attributed to its blend of a random component and a clique-emerging component, which is driven by individuals being tied to default spaces. On the other hand, its performance significantly declined in the high school setting, highlighting limitations in its adaptability. The specific characteristics of our models, particularly the role of the *RWP* component in *STEPS+RWP*, will be the subject of future investigations.

While our study has provided insights into the behavior and characteristics of temporal contact networks, limitations need to be acknowledged. Our current approach of temporarily stacking networks to represent extended time periods does not accurately capture the long-term dynamics of infection spread. However, we believe that our methodology remains valuable for providing insights and deepening our understanding of how confined spaces influence infection dynamics. To improve the accuracy of our models, future studies will need real-world contact data that covers longer periods.

Our parameter optimization strategy, described in Section 3.3.3, aims to replicate SIR properties, contact durations, and edge counts. Future work should also address the temporal distribution of edge occurrences to accurately capture typical events in environments like high schools, offices, or supermarkets (such as rush hours or lunch breaks). Beyond incorporating exact departure times and specific temporal events, future models could also benefit from integrating additional human mobility frameworks that more closely represent the complex behaviors and interactions found in these environments.

## 6. Conclusions

This paper has presented a comprehensive approach to modeling micro-level contact networks through human mobility models, focusing on refining the realism and fidelity of infection spreading in temporal-dynamic networks. By integrating Bayesian optimization for hyperparameter tuning and utilizing network metrics, we have demonstrated the potential of our approach in replicating the infection propagation characteristics of contact networks. By integrating the nuances of different confined spaces, our work can contribute to the overall quality of pandemic simulations and improve the reliability of forecasting models.

The discussion has highlighted the strengths of our methodology, including the capability to optimize HMM parameters using real-world network data, an analysis of network metrics, and the interpretation of optimized model parameters. These advancements pave the way for a more nuanced understanding of how different micro-level encounter models impact the spread of infectious diseases. However, the study also acknowledged limitations, such as the constrained scope of our experiments and the need for broader validation across diverse locations and scenarios. Besides expanding the dataset, exploring additional models, and integrating our approach into larger-scale epidemic simulations with multiple locations, future work could incorporate temporal events specific to certain location types, such as lunch times in offices and peak hours in supermarkets, to better reflect real-world dynamics.

In conclusion, this paper contributes to the field of epidemiological modeling by offering a framework for generating contact networks that align with infection propagation characteristics observed in temporal contact networks constructed from real-world contacts. Our model can be particularly useful for regions or countries where such detailed data are unavailable, providing valuable insights through simulated scenarios. Our work fills a gap by providing a method to model infection dynamics in confined spaces, thereby supporting larger-scale epidemic simulations and forecasting models.

## Figures and Tables

**Table 1:** **Parameters used for different locations and network properties.** ρ is the density [m^2^/node], and β represents the SIR transmission rate. $N_{V}$ denotes the number of nodes and $N_{E}$ represents the number of edges in the original real-world network.

Parameter	High School	Primary School	Office	Supermarket
ρ [m^2^/node]	2.0	2.0	10.0	10.0
β	0.007	0.0013	0.013	0.075
$N_{V}$	327	242	217	539
$N_{E}$	47,300	60,623	12,162	6660

**Table 2:** **Comparison of parameters resulting from Bayesian optimization procedure for *STEPS* and *STEPS+RWP***.

Location	STEPS			STEPS+RWP			
	Npps	k	$\alpha_{t_{p}}$	Npps	k	$\alpha_{t_{p}}$	$t_{p,\max}$ **[s]**
High school	27	4.388	0.421	21	8.128	0.120	3588
Primary school	39	9.974	0.613	22	7.870	0.520	3600
Supermarket	20	2.387	2.887	24	9.161	2.172	24
Office	24	2.881	0.768	40	5.120	0.346	317

**Figure A1:**
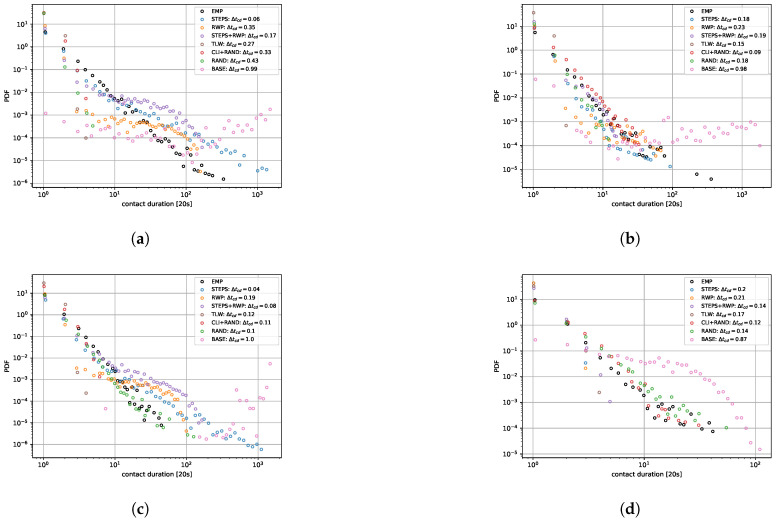
**Contact duration across different locations.** The y-axis represents the probability density function (PDF), and the x-axis indicates contact duration. The legend identifies each model, along with the difference in contact duration $\Delta t_{cd}$ compared to the empirical network (EMP). (**a**) High School; (**b**) Office; (**c**) Primary School; (**d**) Supermarket.

**Figure A2:**
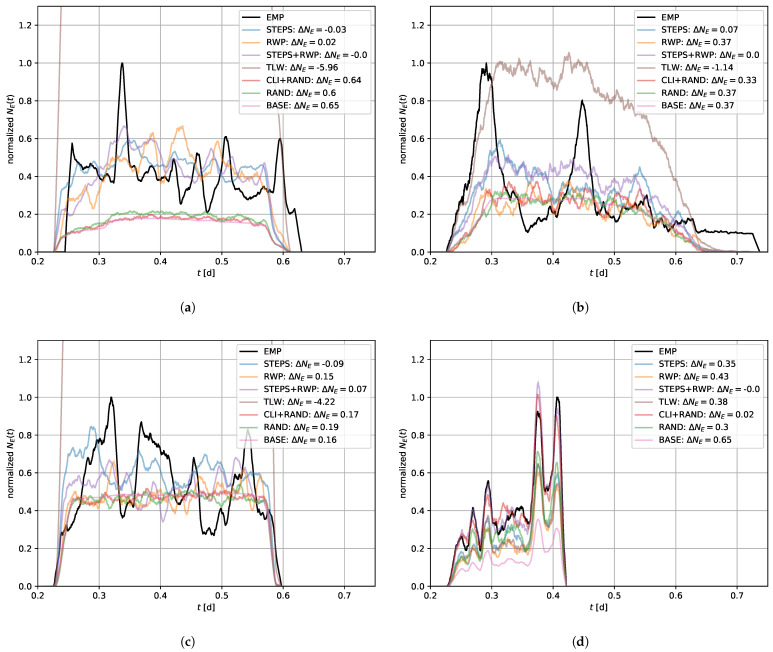
**Normalized edge counts across high school, primary school, office, and supermarket networks.** The y-axis shows the normalized edge count ($\Delta N_{E}\left(t\right)$), the x-axis depicts the time in days (t[d]). The legend identifies the different models along with the overall difference in edge counts ($\Delta N_{E}$). All depicted edge counts are smoothed over 50 time steps. (**a**) High School; (**b**) Office; (**c**) Primary School; (**d**) Supermarket.

**Figure A3:**
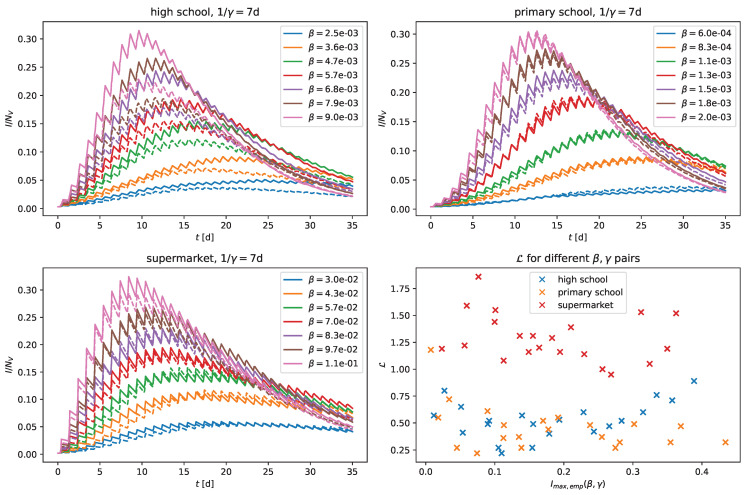
**Impact of *β* and *γ* values on *STEPS’s* performance.** Results are shown for the high school, primary school and the supermarket scenarios. Each line shows the infection curve of the reference network for a specific β value (the exact beta values are provided in the legend). The dotted lines represent the infection curves of the generated networks. The plot at the right bottom shows the cost values for 21 β–γ pairs, depicted for each of the three locations.

## Data Availability

The socio-pattern data used in this study are freely available and can be accessed at the following repository: http://www.sociopatterns.org/datasets/ (accessed on 30 July 2024). The supermarket data used in this study are available upon request due to legal restrictions.

## Appendix A

### Appendix A.1

**Table A1:** **Parameters used for HMMs and naive encounter models.** Depicted parameter ranges were used for Bayesian optimization.

Parameter	Methods	Value Range	Short Description
*w*	*BASE*	[0, 0.01]	Contact intensity, probability to propagate virus within one TU is βw.
$p_{RAND}$	*RAND*, *CLI+RAND*	[0.00001, 0.01]	If an edge is possible, i.e., both nodes are present at location at the same time, how likely it is that this edge occurs.
$\alpha_{cd}$	*RAND*, *CLI+RAND*	[0.1, 10.0]	Shape of the Pareto distribution that contact durations are drawn from.
$p_{CLI}$	*CLI+RAND*	[0.0001, 0.01]	Probability for node to change space per TU.
μ	*CLI+RAND*	[5 TU, 720 TU]	Mean of normal distribution that time spent at non-default location is drawn from.
σ	*CLI+RAND*	[1 TU, 100 TU]	Variance of normal distribution that time spent at non-default location is drawn from.
Npps	*CLI+RAND*, *STEPS*, *STEPS+RWP*	[1, 40]	Number of people that have one space as their default space.
*k*	*STEPS*, *STEPS+RWP*	[1.1, 10.0]	How strong nodes are attached to their default space and its close surroundings.
$\alpha_{t_{p}}$	*STEPS*, *STEPS+RWP*	[0.1, 10.0]	Shape of the Pareto distribution that pause times are drawn from.
$U(v_{STEPS,min},v_{STEPS,max})$	*STEPS*, *STEPS+RWP*	0.83 ms^−1^, 3.2 ms^−1^, fixed values [33]	Uniform distribution that travel speed between spaces is drawn from.
$U(v_{RWP,min},v_{RWP,max})$	*RWP*, *STEPS+RWP*	0.1 ms^−1^, 1.0 ms^−1^, fixed values [33]	Uniform distribution that travel speed within spaces is drawn from.
$U(0s,t_{p,max})$	*RWP*, *STEPS+RWP*	[10 s, 1 h]	Upper limit of uniform distribution that pause times are drawn from. Lower limit is always 0 s.
$\alpha_{t_{p},trunc}$	*TLW*	[−10.0, −0.1]	Shape of truncated power law that pause times are drawn from.
$H_{t_{p}}$	*TLW*	[1 s, 1 h]	Maximum value of truncated power law that pause times are drawn from.
$\alpha_{l,trunc}$	*TLW*	[−10.0, −0.1]	Shape of truncated power law that flight lengths are drawn from.
$H_{l}$	*TLW*	[10 m, 100 m]	Maximum value of truncated power law that flight lengths are drawn from.

### Appendix A.4

The variation in the infection parameter β and the recovery parameter γ impacts the spread of infection. To ensure that our models can adapt to various β, we tested seven different β values for each location, exploring ranges that result in up to a 40 percent infection peak. The models demonstrated better adaptability to different β values in locations where the results were generally more favorable (see Figure 1). Notably, there was some deviation in the high school scenario, particularly at higher β values. We observed similar results for the office network. Additionally, the L was plotted against the maximum infection peak in the lower right part of the figure, showing results for 21 different β-γ pairs for each location. The depicted β ranges were combined with three different γ values, 1/γ= 4 days, 1/γ= 7 days, and 1/γ= 10 days, to reflect recovery on average after 4, 7, and 10 days. We observed only minor fluctuations around the median cost, indicating good adaptability of our model. However, these are preliminary findings, and further investigation into β–γ values and the underlying reference network’s impact on model performance is necessary.

## References

[1] Dalziel B.D., Pourbohloul B., Ellner S.P. (2013). Human mobility patterns predict divergent epidemic dynamics among cities. Proc. R. Soc. B Biol. Sci..

[2] Balcan D., Gonçalves B., Hu H., Ramasco J.J., Colizza V., Vespignani A. (2010). Modeling the spatial spread of infectious diseases: The GLobal Epidemic and Mobility computational model. J. Comput. Sci..

[3] Eubank S., Guclu H., Anil Kumar V.S., Marathe M.V., Srinivasan A., Toroczkai Z., Wang N. (2004). Modelling disease outbreaks in realistic urban social networks. Nature.

[4] Glass L.M., Glass R.J. (2008). Social contact networks for the spread of pandemic influenza in children and teenagers. BMC Public Health.

[5] Liu F., Li X., Zhu G. (2020). Using the contact network model and Metropolis-Hastings sampling to reconstruct the COVID-19 spread on the “Diamond Princess”. Sci. Bull..

[6] Firth J.A., Hellewell J., Klepac P., Kissler S., Kucharski A.J., Spurgin L.G. (2020). Using a real-world network to model localized COVID-19 control strategies. Nat. Med..

[7] Thurner S., Klimek P., Hanel R. (2020). A network-based explanation of why most COVID-19 infection curves are linear. Proc. Natl. Acad. Sci. USA.

[8] Diallo D., Schönfeld J., Hecking T., Cherifi H., Rocha L.M., Cherifi C., Donduran M. (2024). Travel Demand Models for Micro-Level Contact Network Modeling. Complex Networks & Their Applications XII.

[9] Hinch R., Probert W.J.M., Nurtay A., Kendall M., Wymant C., Hall M., Lythgoe K., Cruz A.B., Zhao L., Stewart A. (2021). OpenABM-Covid19—An agent-based model for non-pharmaceutical interventions against COVID-19 including contact tracing. PLoS Comput. Biol..

[10] Kerr C.C., Stuart R.M., Mistry D., Abeysuriya R.G., Rosenfeld K., Hart G.R., Núñez R.C., Cohen J.A., Selvaraj P., Hagedorn B. (2021). Covasim: An agent-based model of COVID-19 dynamics and interventions. PLoS Comput. Biol..

[11] Kühn M.J., Abele D., Mitra T., Koslow W., Abedi M., Rack K., Siggel M., Khailaie S., Klitz M., Binder S. (2021). Assessment of effective mitigation and prediction of the spread of SARS-CoV-2 in Germany using demographic information and spatial resolution. Math. Biosci..

[12] Klise K., Beyeler W., Finley P., Makvandi M. (2021). Analysis of mobility data to build contact networks for COVID-19. PLoS ONE.

[13] Müller S.A., Balmer M., Charlton W., Ewert R., Neumann A., Rakow C., Schlenther T., Nagel K. (2021). Predicting the effects of COVID-19 related interventions in urban settings by combining activity-based modelling, agent-based simulation, and mobile phone data. PLoS ONE.

[14] Hekmati A., Luhar M., Krishnamachari B., Matarić M. (2022). Simulating COVID-19 classroom transmission on a university campus. Proc. Natl. Acad. Sci. USA.

[15] Lee B., Lee M., Mogk J., Goldstein R., Bibliowicz J., Brudy F., Tessier A. (2021). Designing a Multi-Agent Occupant Simulation System to Support Facility Planning and Analysis for COVID-19. Proceedings of the Designing Interactive Systems Conference 2021.

[16] Ying F., O’Clery N. (2021). Modelling COVID-19 transmission in supermarkets using an agent-based model. PLoS ONE.

[17] Reveil M., Chen Y.H. (2022). Predicting and preventing COVID-19 outbreaks in indoor environments: An agent-based modeling study. Sci. Rep..

[18] Bettstetter C., Hartenstein H., Pérez-Costa X. (2004). Stochastic properties of the random waypoint mobility model. Wirel. Netw..

[19] Shin R., Hong S., Lee K., Chong S. On the Levy-walk nature of human mobility: Do humans walk like monkeys?. Proceedings of the IEEE INFOCOM 2008.

[20] Nguyen A.D., Sénac P., Ramiro V., Diaz M. (2011). STEPS-an approach for human mobility modeling. Proceedings of the NETWORKING 2011: 10th International IFIP TC 6 Networking Conference.

[21] Holme P., Saramäki J. (2012). Temporal Networks. Phys. Rep..

[22] Leitch J., Alexander K.A., Sengupta S. (2019). Toward epidemic thresholds on temporal networks: A review and open questions. Appl. Netw. Sci..

[23] Sharkey K.J., Fernandez C., Morgan K.L., Peeler E., Thrush M., Turnbull J.F., Bowers R.G. (2006). Pair-level approximations to the spatio-temporal dynamics of epidemics on asymmetric contact networks. J. Math. Biol..

[24] Rocha L.E.C., Liljeros F., Holme P. (2011). Simulated Epidemics in an Empirical Spatiotemporal Network of 50,185 Sexual Contacts. PLoS Comput. Biol..

[25] Hethcote H.W. (2000). The Mathematics of Infectious Diseases. SIAM Rev..

[26] Vestergaard C.L., Génois M. (2015). Temporal Gillespie Algorithm: Fast Simulation of Contagion Processes on Time-Varying Networks. PLoS Comput. Biol..

[27] Mastrandrea R., Fournet J., Barrat A. (2015). Contact patterns in a high school: A comparison between data collected using wearable sensors, contact diaries and friendship surveys. PLoS ONE.

[28] Stehlé J., Voirin N., Barrat A., Cattuto C., Isella L., Pinton J.F., Quaggiotto M., Van den Broeck W., Régis C., Lina B. (2011). High-resolution measurements of face-to-face contact patterns in a primary school. PLoS ONE.

[29] Génois M., Barrat A. (2018). Can co-location be used as a proxy for face-to-face contacts?. EPJ Data Sci..

[30] Tanis C.C., Nauta F.H., Boersma M.J., Van der Steenhoven M.V., Borsboom D., Blanken T.F. (2022). Practical behavioural solutions to COVID-19: Changing the role of behavioural science in crises. PLoS ONE.

[31] Cattuto C., Van den Broeck W., Barrat A., Colizza V., Pinton J.F., Vespignani A. (2010). Dynamics of person-to-person interactions from distributed RFID sensor networks. PLoS ONE.

[32] Akiba T., Sano S., Yanase T., Ohta T., Koyama M. Optuna: A next-generation hyperparameter optimization framework. Proceedings of the 25th ACM SIGKDD International Conference on Knowledge Discovery & Data Mining.

[33] Mboup D., Diallo C., Cherifi H. (2022). Temporal Networks Based on Human Mobility Models: A Comparative Analysis With Real-World Networks. IEEE Access.

